# Proposal of a Holistic Model to Support Local-Level Evidence-Based Practice

**DOI:** 10.1100/tsw.2010.144

**Published:** 2010-08-03

**Authors:** Said Shahtahmasebi, Luis Villa, Helen Nielsen, Hilary Graham-Smith

**Affiliations:** ^1^ School of Health, Wintec, Hamilton, New Zealand; ^2^ The Good Life Research Centre Trust, Rangiora, New Zealand; ^3^ Pinnacle Group Limited, Hamilton, New Zealand

**Keywords:** local knowledge, evidence-based practice, public health, knowledge transfer

## Abstract

In response to a central drive for evidence-based practice, there have been many research support schemes, setups, and other practices concentrating on facilitating access to external research, such as the Centre for Evidence Based Healthcare Aotearoa, the Cochrane Collaboration, and the York Centre for Reviews and Dissemination. Very little attention has been paid to supporting internal research in terms of local evidence and internal research capabilities. The whole evidence-based practice movement has alienated internal decision makers and, thus, very little progress has been made in the context of evidence informing local policy formation. Health and social policies are made centrally based on dubious claims and often evidence is sought after implementation. For example, on record, most health care practitioners appear to agree with the causal link between depression and mental illness (sometimes qualified with other social factors) with suicide; off the record, even some psychiatrists doubt that such a link is applicable to the population as a whole. Therefore, be it through misplaced loyalty or a lack of support for internal researchers/decision makers, local evidence informing local decision making may have been ignored in favour of external evidence. In this paper, we present a practical holistic model to support local evidence-based decision making. This approach is more relevant in light of a new approach to primary health care of “local knowledge” complementing external evidence. One possible outcome would be to network with other regional programmes around the world to share information and identify “best” practices, such as the “Stop Youth Suicide Campaign”(www.stopyouthsuicide.com).

## INTRODUCTION

Although decision making based on local health care needs is not a new phenomenon (e.g., see [1,2,3,4,5,6,7,8]), evidence-based practice appears to be subscribing to a movement that involves understanding the importance of generating "local knowledge" in order to complement external research (national and/or international) (e.g., [1,9,10,11,12,13,14,15,16,17,18,19,20]). With the New Zealand

Government's new policy on primary health care provision[21], primary health care groups find it necessary to have access to evidence specific to the locality they serve in order to critique or backup and complement other available evidence. For example, in a recent New Zealand government policy[21], at least some local and regional primary and community health care agencies have made the generation of "local knowledge" a priority. Furthermore, "local knowledge" forms part of their new Quality Plan (Accreditation, Periodic System Review, Patient Safety, Evaluation); evaluation will be operational research aiming also at the dissemination of findings when relevant.

Generating local knowledge will give the health care providers the same or similar challenges to seek and generate evidence externally[22,23]. These challenges are related to understanding what constitutes evidence and that can be translated into *critical* assessment and use of research results, a good understanding of research, a good understanding of methodologies (research and analytical methods), issues relevant to IT and ICT and data storage, and so on. For example, in a study[23], 29 barriers to using research in practice were identified and these were broadly categorised into four groups: (1) accessibility of research, (2) research awareness and critical skills of the nurse, (3) organisational setting, (4) quality of research available.

In this discussion paper, we propose a model designed to reinforce the foundation for local knowledge to inform the process of evidence-based decision making at a local level.

## BACKGROUND

Over the last couple of decades, there has been a significant drive for evidence-based practice and a push for policy to be informed by evidence. With the evidence-based practice movement taking shape in the health sector in the late 1980s and early 1990s, randomised control trials (RCTs) became the gold standard. By the mid 1990s, the phrase "evidence based" became synonymous with RCTs and the two were used interchangeably. The emphasis on evidence-based practice shifted attention from appropriate rigorous research methodology to the practice of RCTs. On the other hand, RCTs have a role to play in assessing the effect of a particular treatment in a population, but, in areas where health outcomes are influenced by human behaviour, such as dynamics of disease development, RCTs have no or a limited role to play (e.g., see [24,25,26]). It is plausible that RCT-type activities may have received a higher proportion of resources and funding, thus neglecting research programmes and activities that focus on local knowledge and relevant health issues.

Evidence-based practice has remained a hot topic for clinicians, public health practitioners, planners, and the public. There are now frequent workshops on how to practice and teach it, and centres for evidence-based practice have been established globally (e.g., see [27]), such as the Centre for Evidence Based Healthcare Aotearoa, the Cochrane Collaboration, and the York Centre for Reviews and Dissemination. Evidence-based practice branched off to become more specific and more discipline focused, e.g., evidence-based medicine or evidence-based nursing, and unsurprisingly led to the establishment of specific evidence-based centres, e.g., [28,29,30,31,32,33]. However, most of these centres still concentrate on clinical decision making and, thus, RCTs, systematic reviews, and meta-analyses, or all of the above, are their main core activities.

Although such centres provide invaluable services to support evidence-based practice, their main activity has been related to improving access to external evidence[34]. In other words, there is very little difference between centres of evidence-based medicine and evidence-based nursing; they support or carry out RCTs, systematic reviews, and meta-analyses of existing published research. For example, the Centre for Evidence Based Healthcare Aotearoa's mission statement[29] is "to improve the effectiveness of clinical practice and positively influence healthcare outcomes by synthesising evidence to improve quality and effectiveness in healthcare." These types of activities will be beneficial to experienced and busy medical researchers and health practitioners to inform their process of clinical decision making. However, it is not clear how the less-experienced researchers, be it in planning, clinical, or management, are able to incorporate the support for internal evidence-based practice. For example, in a study, Funk[23] broadly categorised barriers to using research into four groups (see Introduction), three of which are concerned with the dynamics of research and only one category to the accessibility to research.

The main emphasis in gathering evidence has been on research. For evidence to be reliable, research must be academically rigorous and conducted appropriately with the relevant study design and analysis tools. Very few evidence-based centres globally have considered supporting "research" as a main activity to promote and support evidence-based practice, yet, in general, organisational and political emphasis has been on evidence, i.e., research to inform the process of decision making. To this end, evidence-based activities have been more prescriptive toward the "gold standard" and/or systematic reviews, and meta-analyses or other tools associated with generating evidence, such as numbers needed to treat (NNT), sensitivity and specificity, relative risk, and odds ratios, are on their own of meagre value to inform policy. Design, delivery, and evaluation of health care services involve different activities and require a different class of evidence (e.g., see [35,36,37,38]).

For example, in response to the New Zealand Government's initiative "Sooner, better, more convenient"[21], eligible primary health care providers have or are in the process of developing their own initiatives. All of these activities, including development, implementation, and evaluation, will need to be informed by reliable evidence. It is imperative to support research by health care practitioners for both internal (professional's own expertise) and external (published national and international research) evidence. At the time of writing, the authors were aware of five Primary Health Organisations across the Midlands Region (North Island) that had finalised an Expression of Interest to be presented to the Ministry of Health to deliver "Sooner, better, more convenient" primary health care across the said region. The proposal includes the design and implementation of a "Periodic Service Review" (PSR) for identifying the standard of excellence for the provision of patient-centred health care. The aim is to develop a robust process for monitoring and the evaluation of health outcomes. The PSR will collect and process data on a regular basis, but will need to be complemented with an equally robust research component in order to produce evidence-based health intelligence.

The main issue for the providers is that their core activities involve the delivery of care and very little research. On the other hand, it is expected that the delivery of care and planning of services must be based on research. However, all groups experience barriers to critical assessment and use of evidence (e.g., see [34]). Sackett and colleagues[27] suggest that evidence-based practice ought to be the integration of clinical expertise and the best external evidence. In the context of evidence, "clinical" is taken to refer to the expertise of the health care professional that is more relevant to primary or public health care development. In the next section of this paper, we propose a model to support evidence-based practice at an organisational level, to develop the organisation's skills and expertise in doing and using research, and to evaluate the generated local evidence critically.

## THE "EVIDENCE"

Having improved access to evidence, the issue must be to ensure the critical utilisation of the evidence. However, it is plausible to assume that the average decision maker may, in the first instance, be interested and rely on the conclusions and recommendations in published evidence. As an example, the Cochrane Database was searched using the search term "EBM" followed by "psychosocial problems", which yielded eight and 51 hits, respectively. The eight records[39,40,41,42,43,44,45,46] from the first search and 20% of the records from the second search (about ten records)[47,48,49,50,51,52,53,54,55,56] were briefly examined for their conclusions and recommendations. The majority of the reviews appeared to indicate weaknesses and highlighted limitations, including small group numbers and insufficient statistical power, and suggest that larger and better RCT designs with higher statistical power are needed. Despite the limitation caveat, some appeared to suggest some treatment benefits. And where the treatment involved drugs, only one study discussed side effects, but separately and independent of their recommendation.

Most users in the field rely on the researchers' interpretations of the appropriateness of the "evidence". The danger is, of course, allowing additional risks to the public through a decision/policy based on available, but insufficient, evidence from RCTs. In order to be able to utilise evidence critically for policy/decision making, the user must be familiar with research methods, substantive issues, and analytical methods, in order to interrogate the evidence that s/he had accessed appropriately. Experience suggests otherwise. For example, one study[57] asserts: "Significantly reduced rates of further self-harm were observed for depot flupenthixol vs. placebo in multiple repeaters (0.09; 0.02 to 0.50), and for dialectical behaviour therapy vs. standard aftercare (0.24; 0.06 to 0.93)." A statistical significance on its own may not necessarily constitute evidence of a treatment effect[58]. In the above case, the wide confidence intervals render the odds ratios useless in the context of any evidential value. Indeed, the authors concluded that further larger trials were needed.

Another interesting issue that arises is the behaviour of the evidence-based practitioners. Specifically, would they accept the evidence at face value? Or exclude the evidence from the policy formation process? Or would they allow the evidence, but allow for associated risks? Or would they continue to consult the "evidence"? Or seek evidence from research methodology appropriate to the issue under investigation.

## THE PROPOSED MODEL

The dynamics of decision making as a process are not fully studied (e.g., see [59]) and a historical discussion is beyond the scope of this paper. Unlike manufacturing entities where research drives product development and production, health and social care delivery is directly linked to policy development and political decision making. These are then translated by the health care system into resources and funds needed to deliver the policy. The current environment that insists on research-based decisions has placed pressure on organisations that have traditionally had research carried out by a member university to become researchers and/or research users themselves. On the one hand, research in itself does not constitute evidence; it is a systematic form of enquiry and, rightly so, it often raises more questions requiring further research. On the other hand, there is a lack of expertise and experience in (1) developing organisational (local) research, and (2) critical evaluation of the relevance of published (external) research. In recent years, the common adopted approach has been to claim a decision was made based on the "best" *available* evidence *at the time*.

As discussed above in the Background, issues related to the dynamics of research are more of a barrier than accessibility to research. Therefore, support schemes for evidence-based practice may consider aligning with the following principles:

- Research that responds to fundamental questions that need an answer in order to facilitate better delivery of services, curative or preventative, in the primary care sector (or in the interface between primary and secondary)
- Research that can be scoped, undertaken, analysed, and finalised in a reasonable period of time
- The findings of which can be immediately applied to the implementation of new projects, the delivery of current programmes, and/or used for strategic planning purposes

Any model that subscribes to the aim of improving access to external research will not have an impact on generating the appropriate evidence, nor to the development of an evidence-based research culture.

## CONCEPTUALISATION

It is assumed that most universities and, in particular, community-based educational establishments, such as polytechnics, have an established collaborative relationship with community care and health care providers. Anecdotally, this relationship tends to be academic vs. practice, where academic expertise is sought when needed and vice versa. To facilitate and increase collaboration from inception of a research idea to completion of a research project, some educational establishments have introduced a voucher system where community agencies may apply for a voucher that could then be used to purchase academic expertise. Some of such activities appear to have developed collaborative and joint projects. For example, the School of Health at Wintec (Waikato Institute of Technology) has established relationships with a number of regional health care agencies and has developed a number of collaborative proposals for external funding. From this relationship and the authors' past experiences, it became clear that research experience is necessary in order for the community to respond effectively to the Government's initiatives and proactively incorporate evidence in health care service development.

It was also clear that it is beyond the scope of a care service provider to become a research/academic organisation. To be a research user, research experience is necessary to enable a critical evaluation of the available evidence that may not be viewed as being directly relevant to local issues. It is a reasonable assumption that it is desired that local policy making is based on local evidence researched and developed locally.

There are several important issues inherent to evidence-based practice that will be the difference between successful and unsuccessful evidence-based practice. Quite apart from issues relevant to research, including funding and resources distribution, the following issues are major contributors to "good" or "poor" practice:

- Politicisation of research and science
- Ability to evaluate "evidence"
- Ownership of evidence
- Ability to translate findings into service and programme improvements for better clinical and population outcomes

Without any reference to the literature, the first point can be assumed by a natural deduction or simply by implication. As mentioned in the Background section, evidence-based practice is encouraged to be an integral part of decision making processes, not only in health and social policy making, but also in all other aspects of the political process, such as economy and environment. It is therefore reasonable to assume that research and science (as evidence) will be subject to politics due to human behaviour that will no doubt affect the distribution of funds and resources. It is no surprise that a quick search of Google Scholar using the search term "politicisation of research and science" yielded over 20,000 documents, e.g., [59,60,61,62,63,64,65,66,67,68,69,70].

The question is not whether the politicisation of research and science is inevitable, the question is: Why is "politics" often ignored in studies of health and social care? The reverse adds the risk of decision makers/politicians hiding behind science and experts (e.g., see [61]).

A major outcome of the political effects on the research scene, over and above the politics of funding, such as distribution of research funds, has been the uncritical use of research. At higher levels, there is some evidence to suggest that in politics, supportive evidence is often sought after a decision has been made (e.g., see [69]). At the practice level, at least in part, the issue is related to the system of education, training, and experience that may or may not enable a critical evaluation of the evidence (e.g., see [66,71]). In this context, with the advancement in information technology, the information is no longer the privilege of those with formal training and is often readily accessible (e.g., via the Internet) to the public. The Internet is an unregulated source; nevertheless, it has played a major role in empowering the public merely through providing access to information. Empowerment in such a way will have a complex impact on behaviour and practitioner-public interactions (e.g., see [65,67]); for example: (1) the impact on public perceptions and expectations from an apparent inability by practitioners to evaluate accessed information critically and convey research results, (2) the impact on behaviour from an *uncritical* individualisation of the available evidence.

While the practice of evidence-based decision making may encourage and produce a culture of research "usership", it does not address "ownership" of evidence that plays an important role in local planning, as demonstrated in different areas of planning (e.g., see [72,73,74,75,76,77,78,79]). The process of decision making can be positively influenced through a sense of ownership of the evidence, local participation, and partnerships to complement external evidence.

Therefore, any model of an evidence-based practice initiative must address these relevant issues of research. It is evident that it would not be too difficult to address the second and third issues, i.e., enabling critical evaluation of evidence and creating a sense of ownership. But, progress will be limited and would depend on successfully addressing the first issue. Perhaps the most challenging is the incorporation of the human behaviour (Politics and politics) aspect in the model. Nevertheless, the current political environment suggests that change towards local participation and ownership of policies, although an old idea, might be marking a new political trend (e.g., see [73,78,79]). This may suggest that part of the challenge is being met through Politics (and politics) itself. Therefore, ignoring political aspects, we may set about exploring how to address the issues of research capacity building and ownership of evidence.

## THE SUPPORT UNIT

Most health care–related evidence-based centres appear to service the experienced researcher or research user by providing access to a database of reviews, systematic reviews, and meta-analyses. Participants or stakeholders in planning a health care service for their locality may not have acquired the necessary skills in critically evaluating the nature, quality, and relevance of evidence provided to them. For example, in some settings, it is reported that community health care practitioners may be receiving their information and evidence from their managers, and are often not aware of primary data sources[22]. Harrison and Eaton cite obstacles related to translating research into evidence for the real world[80].

For example, Briggs and colleagues[22] report that in nursing, there is evidence to suggest research findings and systematic reviews do not reach many nurses. Furthermore, the closer the staff members are to providing direct patient care, the less aware they are of such initiatives (also see [81]). The reasons for this failure to access research have been the focus of much study. Briggs and colleagues[22] carried out a comprehensive review of the literature, which revealed numerous papers highlighting the factors that hinder the use of research in nursing, such as accessing a large volume of research information; critical appraisal and library skills of nurses; the academic presentation of research findings and methods of dissemination; and implementation within organisations[82,83,84,85,86,87,88,89].

The evidence points to an organisational lack of research skills and a lack of research culture becoming partly responsible for obstacles to research. In addition, most inter- and between organisational collaborations, such as with an academic institution, are often in the form of a single or a number of specific projects where a university department may be carrying out the research and reporting back to the care provider organisation. However, this practice does not address the organisational research skills and ownership issues. One solution to this problem may be to inject skill and experience into the local organisations in the form of a Mobile Research Support Unit (MRSU). There are several advantages in establishing such a Unit.

The Unit may be operationalised as a venture between local or regional health care organisations and a university or academic partner. Such a partnership will reduce the initial setup investment. Further funds may be raised through grant applications.

In the first instance, the Unit will address the ownership issue, as it will encourage and support practitioners to develop a critical approach to evaluating evidence. Following critical assessment of the external evidence, practitioners will be supported in developing their local evidence requirement into a formal research proposal and plan. Research plans may then be carried out within the organisational parameters. The university partner will provide academic support and the organisation will provide practical experience. This combination will replace the ad hoc and on-project-basis collaborations. Clearly, this approach will lead to the development of many themes and topics to be researched, and therefore increases the chances of raising the external funding to carry them out.

The Unit will act as a hub for “research” and may increase the potential for a research culture, multidisciplinary and collaborative research, and may increase the utilisation of research results from a number of related health care topics, leading to a holistic approach to service planning (e.g., see [59,66]).

Clearly there will be a monitoring element, which is integral to the conceptualisation and will be based on measuring parameters relevant to a research culture, such as quality and quantity of research initiatives and projects, research participation, research outputs, documentation trails leading “practice” to “evidence”, and so on. In addition, the MRSU will need to establish a longitudinal survey of staff research-related behaviour in order to assess the influence of the Unit on the progress of research capacity building.

The staffing of the Unit will be dependent on the resources available, but mainly on the size and the complexity of the participating organisations.

It is recommended that an Advisory Board be elected comprised of senior staff from the stakeholders to support the MRSU. The Advisory Board functions will be developed and decided on by the stakeholders, and will include approval of the MRSU working plan and research projects. The researcher’s role will be to provide input and support for the approved projects. The Unit will provide expert support to all staff to enable them to translate their observations into research question(s), and subsequently develop them into proposals and conduct their research. In other words, the Unit will support the staff so that they may perform relevant and practical research, from inception to the critical interpretation of the evidence. The Unit will help to disseminate the results of the research widely within the primary health care environment and the health sector in general. It is also proposed that funds be administered through the academic institution stakeholder, but for obvious reasons, the MRSU will be based in the health care provider headquarters, and, to provide a mobile research support across the Midland Region to the stakeholders.

## CONCLUSION

As discussed above, there is sufficient evidence to suggest that systematic reviews and meta-analyses results do not reach health practitioners. This argument has been used to develop new initiatives to address the issue. However, such initiatives are often formulated in the same vein, albeit repackaged slightly differently. For example, Briggs and colleagues[22] offer the idea of a bulletin and suggest more success with nurses citing reading/knowing about it. But most research of this type fails to appreciate the quality and methodology of evidence presented by these types of initiatives. Briggs and colleagues[22], suggest that access to the evidence is only one barrier, the remaining majority of barriers are relevant to quality, organisation, and other relevant local issues. It is plausible that an arrogance of presumption of difficulties with access to evidence has led to development of schemes that only address “access” to evidence. In other words, researchers fail to explore and understand factors governing access to evidence, e.g., *willingness* to utilise external evidence. Furthermore, researchers need to address the inevitable question: Would improved access lead to the commensurate utilisation of evidence?

On the other hand, the emphasis on evidence-based practice is leading to a new generation of health care service workers that are expected to be researchers or research users. These changes are happening within the traditional health service framework. In other words, changes to parts of a dynamic system, while the overall system parameters remain unchanged, create potential for major conflicts, e.g., inexperienced managers attempting to manage experienced practitioner researchers (e.g., see [90]).

We believe that the MRSU, to some extent, addresses these issues by acting as a hub for research and evidence-based management. The MRSU is conceptualised to go beyond the convention of attempting to improve access to external evidence, training workshops, and so on, and is more practitioner centred. As such, the main functions of the MRSU will be directly related to the research needs of practitioners at the time of a request for support. These may involve assistance with a critical literature review (including systematic reviews), assistance with identifying concerns and/or observations from practice and developing them into a research issue and proposal, or assistance with methodological development for research and analysis. But perhaps the most important function would be to ensure an understanding when dealing with human behaviour; that evidence may only be derived from the application of appropriate methodologies to the issue under study and that there is no gold standard.
